# Learning Unicycling Evokes Manifold Changes in Gray and White Matter Networks Related to Motor and Cognitive Functions

**DOI:** 10.1038/s41598-019-40533-6

**Published:** 2019-03-13

**Authors:** Bernhard Weber, Karl Koschutnig, Andreas Schwerdtfeger, Christian Rominger, Ilona Papousek, Elisabeth M. Weiss, Markus Tilp, Andreas Fink

**Affiliations:** 1 Institute of Psychology, University of Graz, Graz, Austria; 2 Institute of Psychology, BioTechMed Graz, University of Graz, Graz, Austria; 3 Institute of Sports Sciences, University of Graz, Graz, Austria

## Abstract

A three-week unicycling training was associated with (1) reductions of gray matter volume in regions closely linked to visuospatial processes such as spatial awareness, (2) increases in fractional anisotropy primarily in the right corticospinal tract and in the right forceps major of the corpus callosum, and (3) a slowly evolving increase in cortical thickness in the left motor cortex. Intriguingly, five weeks later, during which participants were no longer regularly engaged in unicycling, a re-increase in gray matter was found in the very same region of the rSTG. These changes in gray and white matter morphology were paralleled by increases in unicycling performance, and by improvements in postural control, which diminished until the follow-up assessments. Learning to ride a unicycle results in reorganization of different types of brain tissue facilitating more automated postural control, clearly demonstrating that learning a complex balance task modulates brain structure in manifold and highly dynamic ways.

## Introduction

Our brain orchestrates about 85 billion nerve cells as well as non-neural cells^1^, wired via the utterly exceptional number of a hundred trillions of synapses^2,3^ - an endless playground for intriguing dynamic processes in the brain, known as neuroplasticity including all of its facets and varieties^4^.

Neuroplasticity is certainly among the most fascinating phenomena in the field of neurosciences, especially because it exemplifies the high sensitivity of the brain towards environmental demands. However, despite the large progress that has been achieved in animal and human studies dealing with brain plasticity changes as a result of learning in the past decades, our knowledge about the manifold mechanisms underlying neural changes due to skill acquisition is still at a very early stage. The groundbreaking findings^5^ of altered hippocampi found in experienced London taxi drivers are among the first ones demonstrating that specific daily routines – or in a broader sense learning – alter structural characteristics of the brain. Since then, a considerable number of studies investigating structural brain changes as a result of skill acquisition were conducted in various domains, including second language acquisition and proficiency^6,7^, juggling^8^, the domain of musical expertise^9,10^, and especially also the clinical neurological domain^11^. Furthermore, even a physical leisure activity can induce structural brain changes^12^.

Most of these seminal studies focused on experience-dependent changes in gray matter (GM) volume, while more recent studies in this field are also looking at other imaging parameters such as white matter (WM) microstructure in order to assess the manifold ways of how the brain adapts to environmental demands^13–16^. While these studies provided further important insights into experience-dependent changes of the brain, combined approaches utilizing multimodal imaging parameters within one and the same experimental design are comparatively rare^11,13,16,17^. Particularly the fact that learning processes may alter certain characteristics of GM or WM morphology in different ways, each of them occurring with different shape, strength, and latency^14,18^, clearly indicates the necessity to apply longitudinal multimodal imaging parameters to adequately map the manifold dynamics of the brain’s capacity to adapt to environmental stimulation.

This study took an important step towards this direction. In a longitudinal multimodal neuroimaging study involving a pre- post-test design with a professionally supervised 3-week unicycling training in between, and a subsequent follow-up assessment five weeks after the training, we investigated dynamic neural changes implicated in the learning process of a highly complex balance task. We focused on unicycling since this skill is one of the most complex balance tasks someone can learn as one constantly needs to adjust the balance^19^ both forth-back and left-right. At the same time, one needs to pay attention to the muscle force in the feet, to the right speed and pedal rotation frequency. Finally and equally important, one must be aware of the own spatial movements while sitting on a unicycle, requiring sophisticated and skillful feedback from the senses to the whole body motion to adequately operate a unicycle^20^.

There are several studies investigating the effects of balance training on both structural and functional characteristics of the brain, especially in clinical samples and in samples involving older adults. Sehm *et al*.^21^, for instance, administered a whole-body dynamic balancing task in a sample of patients with Parkinson’s disease over a time period of six weeks. They found improvements in balancing ability, which were correlated with changes in GM in brain regions putatively implicated in the coordination of complex body movements (such as the left inferior parietal cortex, the left ventral premotor cortex, or the left middle temporal gyrus). A recent fMRI study^22^ employed a 5 weeks classical balance training (standing with one leg on different unstable grounds) in older adults and found reduced brain activity in regions associated with postural control, in which typically over-activations with increasing age have been found.

In a sample of adults from the normal population, Rogge *et al*.^23^ found improvements in memory and spatial cognition as a result of a balance training conducted over a time period of 12 weeks. The balance training was also associated with widespread changes in cortical thickness in regions supporting visual and vestibular self-motion perception such as the superior temporal gyrus, visual association cortices, the precentral gyri, or the putamen^24^. Significant structural brain changes were observed even after exercising for only two sessions with a complex whole-body balancing task^16^. Strikingly, that study revealed substantial training-induced increases in GM in frontal and parietal brain regions, accompanied by significant decreases in Fractional Anisotropy (FA) in partly overlapping brain regions. More recently, the same group found that a single balance training session resulted in localized increases in cortical thickness in the motor cortex^25^.

Taken together, these studies provide converging evidence that the brain is highly sensitive to learning a motor related or balance task, even after a very short period of time^25^. What is still not well understood in relevant literature is how different types of brain tissue (such as GM and WM morphology) alter in response to the very same learning task (among the rare exceptions is a longitudinal balance training study^16^). For instance, to which extent are significant training-induced changes in GM morphology (GM volume, cortical thickness) related to corresponding changes in WM integrity? Does a motor skill training differentially affect measures of GM volume and cortical thickness? Does the motor training yield brain changes only in motor brain regions (such as the primary motor cortex or the corticospinal tract), or in regions supporting visual and spatial cognition as well? This multimodal imaging study was designed to address some of these important questions which received comparatively less attention so far. We focused on learning to ride a unicycle since this skill is certainly among the most complex balance tasks and hence very likely to induce manifold structural changes in the brain. Another advantage of this task is that participants with no relevant history/experience in this specific ability (i.e., to ride a unicycle) can be tested, therewith providing perfectly equal starting levels for all participants. In addition, learning to ride a unicycle may also constitute an ecologically highly valid approach to improve balancing ability, therewith increasing task commitment and training motivation. On a more general level, brain changes as a result of unicycling may also add evidence to the nascent field of research investigating the role of different kind physical activity interventions (e.g., aerobic training vs. stretching exercises) on cognitive and brain functions^26^.

Changes in brain structure as a result of the unicycling training were assessed by using T1-weighted images for voxel-based analyses of GM and cortical thickness (CT), and diffusion-weighted images for tensor-based morphometry analyses. It was hypothesized that learning to ride a unicycle affects – along with improvements in behaviorally assessed postural control – different types of brain tissue in several functionally relevant neural circuits, especially in networks supporting motor related functions (such as the motor cortex or the corticospinal tract). Relevant literature further leads us to expect that a complex balance training yields significant structural brain changes in regions supporting cognitive functions (e.g., visual or spatial cognition) as well^16,23,24^. We further expect that each imaging modality elucidates shared and specific dynamic brain properties underlying this highly complex process, therewith providing further important insights into learning-induced plasticity changes of the brain. Taubert and coworkers^27^ moreover noted that it is often unclear whether or to which extent alterations in brain structure are linked with individual learning success. Hence, this study also assessed the performance of unicycling after the training which was linked to training-induced changes in brain structure.

## Results

### Changes in Gray Matter Volume

**Figure 1:**
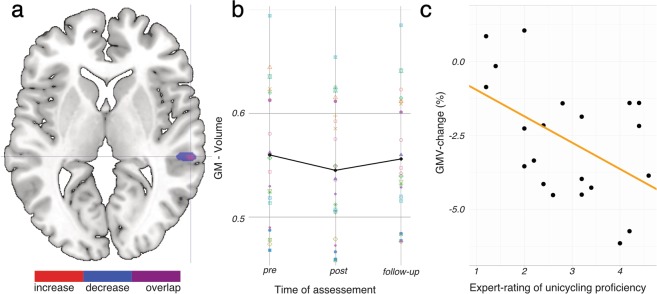
GM volume changes in rSTG between time points of assessment. (**a**) Overlap of decrease and re-increase in rSTG. (**b**) GM changes in rSTG at the individual level. (**c**) Correlation between GMV decrease and unicyling proficiency in rSTG.

Analyses of GM volume revealed focal decreases in the right superior temporal gyrus (rSTG) and in a smaller cluster involving the left parahippocampus right after the three week unicycling-training. Decreases were followed by a significant re-increase in the post-training period. Strikingly, this re-increase was found in the very same region of the rSTG, but topographically more focused. Table 1 and Fig. 1a summarize the brain regions that exhibited training-induced GM volume decreases and re-increases at the follow-up scan. As depicted in Fig. 1b, decreases and subsequent re-increases in GM volume in the rSTG can be clearly observed at the individual level.

**Table 1:** Changes of GM volume over time. Training induced decreases (pre-test > post-test) and re-increases (post-test < follow-up) in GM volume.

Contrast	Region	H	T	k_E_	MNI-coordinates		
					X	Y	Z
**Pre-test > post-test**							
	Superior temporal	R	7.05	586	60	−32	4
	Parahippocampus	L	5.79	49	−26	−42	−14
**Follow-up > post-test**							
	Superior temporal	R	5.95	37	60	−32	4

All results are FWE-corrected (p = 0.05) at voxel-level, k_E_ = cluster size H, hemisphere; L, left; R, right.

In order to examine whether the observed variations in GM volume were linked to the learning success after the unicycling training, a Pearson correlation between unicycling proficiency and training-induced changes in GM volume in the rSTG was computed. We observed a significant negative correlation (GM change_pre-post_ * Performance: *r* = −0.473, *p* = 0.030), indicating stronger GM volume decreases in more proficient individuals (see Fig. 1c).

### Changes in White Matter

**Figure 2:**
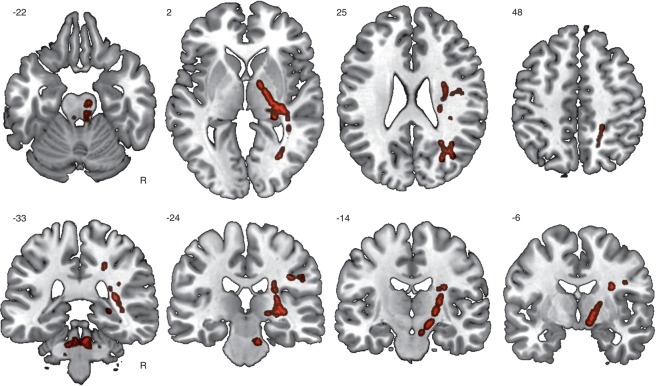
Increases of fractional anisotropy directly after the unicycling training. Results were based on a non-parametric permutation test corrected for multiple comparison (p = 0.05; TFCE, FWE). TFCE, threshold-free cluster enhancement; FWE, family-wise-error.

In contrast to the findings of GM volume changes, we found significant training-induced increases in measures of WM integrity. Specifically, increases in fractional anisotropy (FA) were found in various fiber tracts right after the training, primarily involving tracts linked to motor functions (right corticospinal tract) or visuospatial processes (forceps major of the corpus callosum, a fiber bundle that connects regions of the occipital lobe; see Fig. 2 and Table 2). Increases were also found in axial diffusivity as well as in mean diffusivity (see Fig. S2 and Table S2).

**Table 2:** Changes in WM (FA) between the post-test and the pre-test. (1-p)-values are reported. H, hemisphere; R, right; FWE, family-wise-error.

Region	H	Cluster		MNI-coordinates		
			p-FWE	X	Y	Z
Forceps major	R	1086	0.979	63	59	99
Corticospinal tract	R	901	0.978	76	124	75
		54	0.953	79	101	43
		40	0.957	82	110	54
		22	0.952	80	95	46
Anterior thalamic radiation	R	149	0.964	91	91	44
Corticospinal tract	L	141	0.962	102	96	43
Inferior fronto-occipital fasciculus	R	123	0.957	53	76	67
		56	0.956	57	92	84
		47	0.953	56	63	68
Superior longitudinal fasciculus	R	91	0.964	60	122	96
		85	0.958	49	106	102
		23	0.952	47	120	99
Inferior longitudinal fasciculus	R	24	0.952	50	69	58
		21	0.955	55	63	64

### Changes in Cortical Thickness

**Figure 3:**
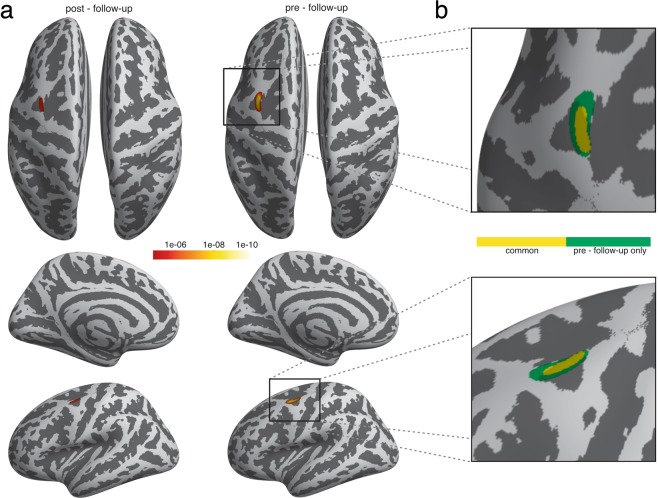
Changes in Cortical Thickness. (**a**) Increase of CT in the left primary motor cortex. (**b**) Enlarged view of overlapping increases; yellow (=common): post-test to follow-up, green: pre-test to follow-up.

In addition to changes in GM volume, we examined changes in CT to map training-induced changes in brain structure with an additional parameter of GM morphology. The analysis revealed a continuous increase of CT in the left superior precentral gyrus across all three time points of assessment. While there was no significant alteration between the pre- and the post-test, the CT in the left precentral gyrus showed a significant increase from the post-test to the follow-up scan, which was even more pronounced between the pre-test and the follow-up scan (see Table 3 and Fig. 3).

**Table 3:** Changes in Cortical Thickness. Increases in CT were observed in the left superior part of the precentral gyrus. Results are FWE-corrected for multiple comparisons (p = 0.05). H, hemisphere; L, left.

Contrast	Region	H	Vertices	t-value
follow up > post-test	Superior part precentral	L	157	5.6
follow up > pre-test	Superior part precentral	L	352	7.7

### Behavioral Changes in Postural Control

**Figure 4:**
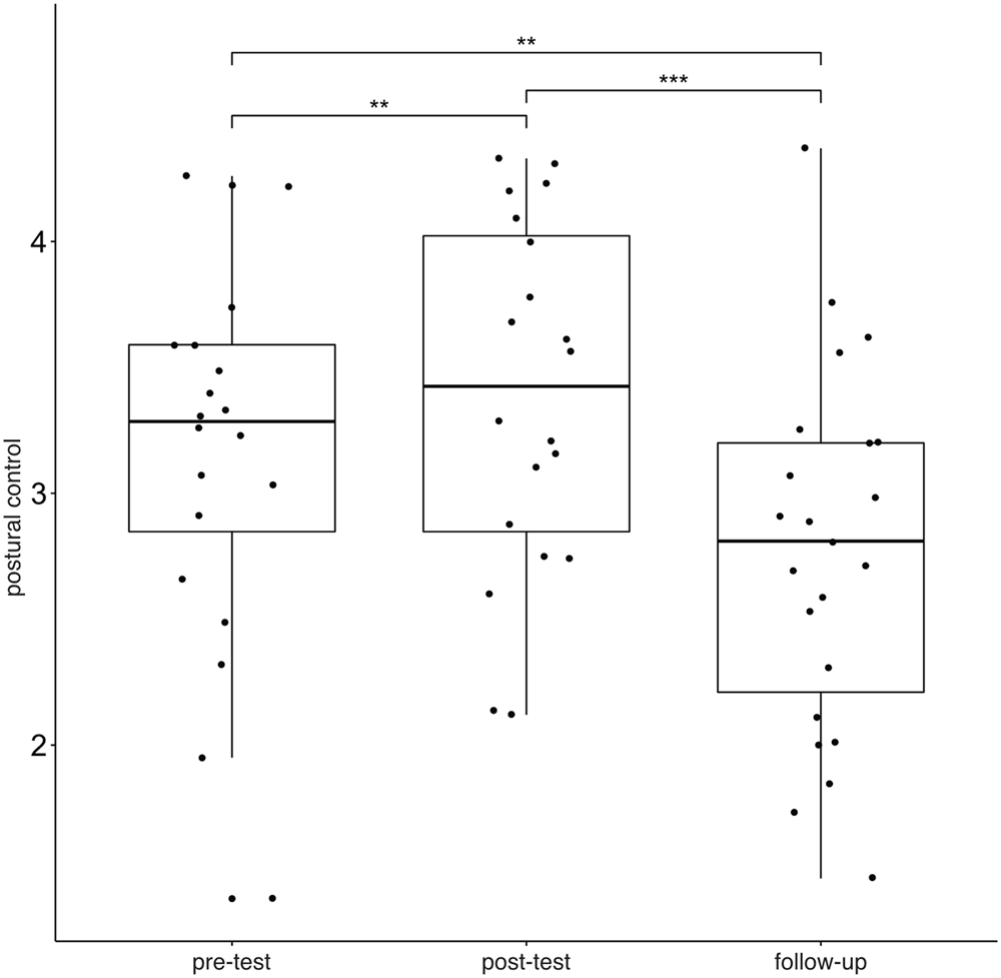
Changes in postural control. Postural control (balance board) of the participants was assessed at three different time points **p  = 0.001, ***p < 0.001.

There was also a significant increase in participants’ postural control after the training (pre-test vs. post-test: t(17) = 3.87, p = 0.001), which significantly decreased until the follow-up assessments (post-test vs. follow-up: t(19) = −8.94, p < 0.001; pre-test vs. follow-up: t(19) = −4.15, p = 0.001; see Fig. 4).

### Multivariate pattern recognition analysis

**Figure 5:**
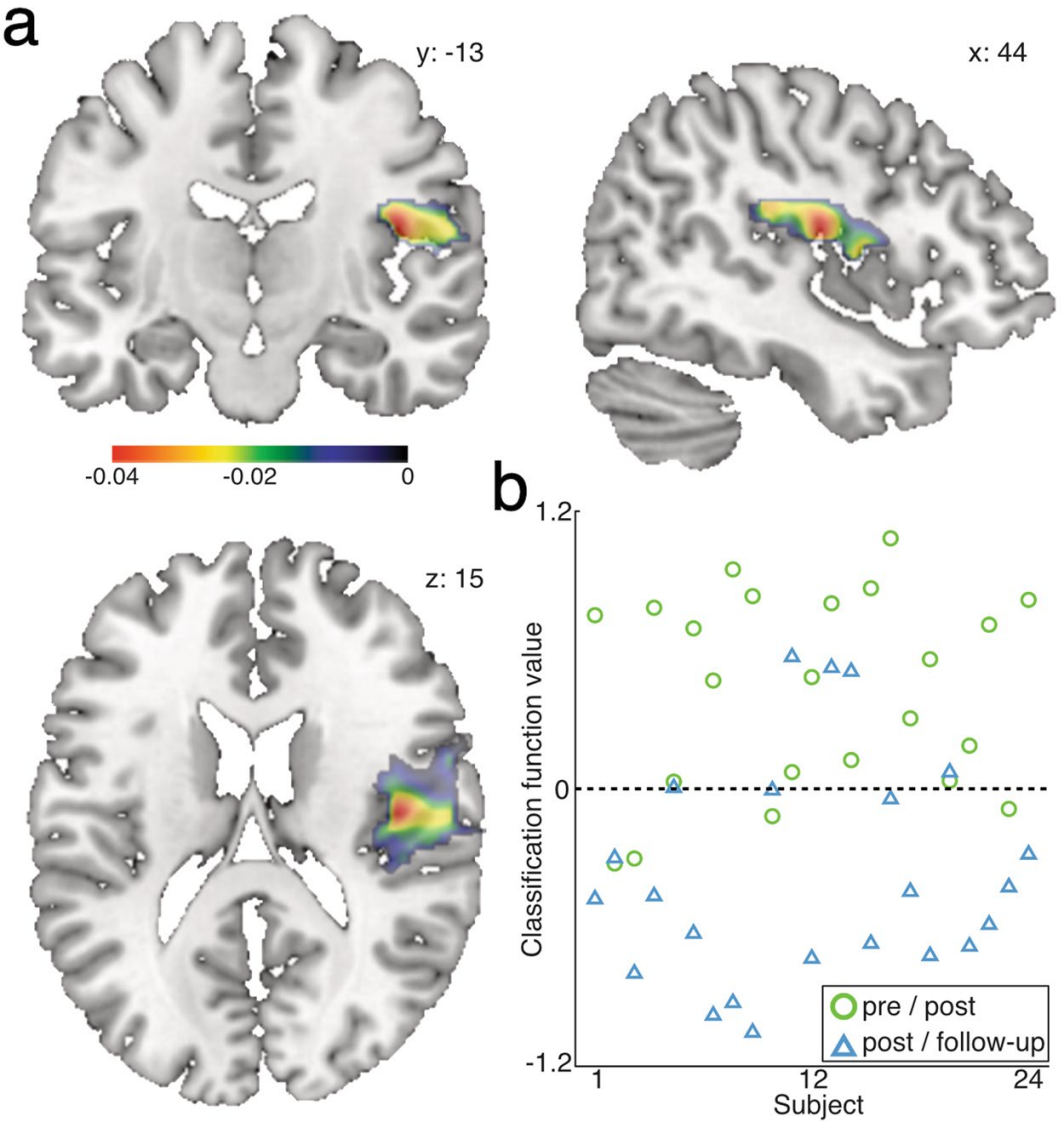
Multivariate pattern classification analysis. (**a**) The weight maps for the area with the greatest predicted power (weight: 72%) is located in the right pars opercularis and the rSTG. Maps are computed by means of a multiple-kernel learning machine based on a pre-parcellated atlas (Automatic Anatomic Labeling) (**b**) Results for the support vector machine classification are shown in the scatter plot. The balanced accuracy is 80.43%. Functional values are plotted for each participant for the learning-period (triangle) and the post-learning period (circle). See also Supplementary Table S1 and Supplementary Fig. S1.

To complement the univariate statistic approach, we further tested the pattern of changes in GM volume before and after the unicycling training in utilizing a completely independent statistical approach. This alternative analysis approach was chosen to further corroborate the robustness of the findings that were obtained via conventional contrasts between the different time points of assessment. For this, we computed difference maps of GM volume of the learning period (post-test – pre-test) and of the post-learning period (follow-up – post-test) respectively. We then applied a multivariate pattern recognition analysis (MVPA) using a support vector machine algorithm and could successfully classify changes in GM volume between the learning period and the post-learning period with a total accuracy of 80.43% (Fig. 5). The regional contribution to the decision function implemented by a multiple kernel learning approach is most prominent in the right rolandic operculum and the rSTG with a predictive power of 72%. Additionally, we conducted the same analysis for FA maps, but here training-induced changes could not be correctly classified (total accuracy: 50%; Supplementary Table S1, Supplementary Fig. S1).

### Control analyses

To provide an additional test that the reported brain changes were due to the unicycling training, the same set of analyses (GM, CT, diffusion-weighted data) was performed for a control group of n = 24 participants (13 females) for which longitudinal structural imaging data for a similar time range were available^28^. As in this study, the participants were scanned at three time points with an interest-interval of about 4 weeks each. They received no unicycling training, neither any other kind of balance training, instead they completed a computerized, 3-week verbal creativity training between the second and the third time point of assessment^28^. For this study, we only used the first two measurements to prevent an interaction with the creativity training.

Analyses in the control group revealed no significant time-related changes across all imaging modalities.

## Discussion

In applying state-of-the-art brain imaging methods involving rigorous statistical thresholds across three different imaging modalities, this study revealed that learning to ride a unicycle was associated with reductions of GM volume, especially in the right STG and in the left parahippocampus. Intriguingly, about five weeks later during which participants were no longer regularly engaged in unicycling, a re-increase in GM in the rSTG was found. At the same time, there was a continuous increase in CT in the left primary motor cortex across the time points of assessment, which, however, was significant only at the follow-up assessment relative to the post- and especially to the pre-test. In addition, diffusion-based analyses revealed increases in FA mainly in the right forceps major and in the right corticospinal fiber tract. These changes in GM and WM morphology were paralleled by significant increases in unicycling performance and by significant training-induced improvements in postural control, which diminished until the follow-up assessments. Taken together, the findings of this study clearly demonstrate that learning a highly dynamic balance task modulates brain structure in manifold and highly dynamic ways.

Learning unicycling was associated with an increase in CT in the primary motor cortex, which has been found to be implicated in various kinds of motor skill learning^29–31^, and to exhibit rather slowly evolving reorganization as a result of motor learning^31^, especially after a significant amount of training^32^. These latter findings may possibly also explain why the observed training-induced increases in CT in this study were only significant at the follow-up test (relative to the pre- and the post-test). The training-induced reductions in GM volume, which were correlated with better unicycling skills after the training, are at first sight somewhat surprising since they seem to run counter the commonly reported GM increases as a result of learning and skill acquisition (e.g.,^6–8,33,34^). It should be noted though, that this study is by no means the only one showing significant reductions in GM in response to training or increased task experience. Similar findings have been reported in motor task learning^35^, whole body balancing^16^, extensive vestibulo-visual stimulation^36^, or long-term musical training^37^. In this study, training-induced GM decreases and subsequent re-increases were most pronounced in the right STG, and there was also a smaller cluster of GM reductions in the left parahippocampus. The latter has consistently been found to be implicated in visuospatial processes including spatial representation and navigation^38^. In a quite similar vein, the rSTG is implicated in processes such as spatial awareness, the exploration of object-related and space-related information, as well as the perception of the visual vertical, also providing information about both the body position relative to external space and motion of one’s body in space^39–44^.

Though this MRI study cannot provide any information about the underlying cellular and molecular mechanisms underlying the observed decreases in GM volume, several findings of this study support the assumption that the observed training-induced GM decreases indicate reorganization of brain tissue facilitating more automated and efficient coordination of voluntary movements and postural control. First, GM reductions in the rSTG were correlated with better unicycling performance after the training, and behaviorally assessed postural control significantly increased after the unicycling training. Second, we found increased FA after the training in the right corticospinal fiber tract and in the right forceps major of the corpus callosum. These changes may reflect more efficient information transfer across cortical pathways, especially in those supporting visuospatial and motor-related functions^45–47^. Taken together these findings hence suggest that successful learning of unicycling was mirrored by structural brain alterations in relevant (i.e., task-specific) brain networks supporting cognitive and motor-related functions.

The multimodal imaging approach utilized in this study also revealed the important finding that training-induced alterations in different types of brain tissue operate on a different time-scale^48,49^. While changes in cortical thickness appear to evolve rather slowly from the pre- to the post-test up to the follow-up test, GM volume decreased after the training and re-increased subsequently. On the contrary, FA increased right after the training and remained stable up to the follow-up test. This pattern of finding clearly indicates that these changes must be driven by different, though potentially highly intertwined biological mechanisms. On a more general level, this finding also provides a strong empirical argument for using multimodal imaging parameters in investigating learning-induced changes in structural and functional characteristics of the brain.

Future research is particularly challenged to elucidate the specific molecular and cellular mechanisms that may underlie the observed learning-induced alterations in brain structure. This especially applies for the observed decreases^18,49^ and re-increases in GM volume. Even if one accepts that learning of a new task is more critical for the brain to change its structure than continued training of an already learned task^34^, possible explanations for these GM alterations are pretty rare. Vaquero and coworkers^37^, for instance, interpreted their findings of increases and decreases in GM in pianists as reflecting some kind of “balance-maintenance” mechanism of the brain (p. 116): While some region show increases in volume, other exhibit decreases in order to compensate for the global volume of the network. The finding of GM decreases in this study, which were paralleled by increases in white matter morphology in overlapping networks, could be interpreted in the pretty same manner.

To the very best of our knowledge, this is the first study showing extensive training-induced decreases in GM with spatially overlapping re-increases after participants were no longer regularly engaged in the training. The particular strength of this study is that we conjointly looked at different brain imaging parameters to assess the manifold dynamic changes as a result of learning a highly dynamic balance task. GM changes and changes in WM microstructure appear to occur more rapidly than training-related changes in CT in the primary motor cortex. This finding also supports the idea that these changes in brain tissue are associated with different molecular and cellular mechanisms (e.g.,^49,50^). There are also some important limitations we should briefly pay attention to. Especially the post-hoc evaluations of control group data, which were taken from a former study involving longitudinal structural imaging data for a similar time range^28^, is suboptimal. Given that these data were obtained within the context of an entirely different research context (creativity training), and especially in view of the fact that the imaging parameters differed from those utilized in this unicycling training study, we deemed it less appropriate to analyze the data of these different studies within one and the same statistical design (2 × 3 design). Related to this, the sample size of this study is comparatively low, which may considerably limit the statistical power of such complex designs. In order to demonstrate that our findings, albeit low sample size and lack of direct control group, are robust and specific to the unicycling training, we kept the stringency level of the analyses of imaging data at the most conservative extent. The following findings of this study strongly support the robustness and specificity of our findings. We found a significant decrease in GM volume in the rSTG and a subsequent re-increase at the follow-up test in exactly that region. Strikingly, the pattern of GM decreases and subsequent re-increases was not driven by some participants (e.g., by some outliers), but was evident in virtually every single participant. Also, decreases in GM from the pre- to the post-test and the subsequent re-increases at the follow-up test were perfectly mirrored by the findings concerning postural control, indicating a training-induced increase at the post-test, and a decrease at the follow-up test. Critically, the significant correlation with task performance involving the rSTG clearly indicated that GM decreases in the rSTG are linked to variations in unicycling performance. And finally, the findings in the other imaging modalities (cortical thickness, diffusion-weighted data) further corroborate the robustness and the specificity of the findings since they were found in relevant networks/brain regions implicated in such a complex motor ability. We hope that the findings reported in this study stimulate intertwined research studies combining brain imaging, histology, and molecular sciences to gain interdisciplinary insights into the manifold macro- and microscopic mechanisms underlying learning-induced plasticity changes of the brain.

## Materials and Methods

### Participants

Twenty-three (13 women) right-handed (as determined by self-report) adults (about 60% students) in the age range between 20 and 51 years (*M* = 30.42, *SD* = 9.14) participated in this study. None of the participants reported any history of medical, psychiatric, or neurological disorders. As an important inclusion criterion, participants were required to be complete beginners in unicycling. All participants gave written informed consent prior to the experiment. For their participation in the MRI-scans, participants received an expense allowance or alternatively course credit. This study was approved by the local ethics committee of the University of Graz, Austria.

### Study Design and Procedure

Changes in structural brain characteristics as a result of learning unicycling were investigated in a pre- post-test design with the three-week unicycling training in between, followed by an additional assessment five weeks after the post-test. The initial MRI-scans were completed prior to the first training session. Right after the third week of training, the post-MRI scans were carried out. At each time point of assessment, MRI-scans including T1-weighted and diffusion-weighted imaging parameters were performed. In addition, we also assessed participants’ postural control and unicycling performance.

### Unicycling-Training

The unicycling training was organized over a time period of three weeks, involving guided group training sessions lasting three hours once per week and private training sessions of the participants. Specifically, six semi-professional unicyclists (three women) instructed the participants in basic principles of unicycling. They especially pointed out (upright) posture, pedal frequency as well as body-balance and helped to avoid beginner’s mistakes.

### Assessment of Task Performance

To assess participants’ unicycling proficiency, participants had to complete an indoor unicycle ride along a corridor over a distance of 25 m in a bidirectional manner. Participants performed this unicycle ride twice: The first assessment of participants’ unicycling skills directly followed the three-week training period (post-test MRI scans), the second assessment was realized five weeks later at the MRI follow-up assessment session. Participants were videotaped, and five experienced unicycling instructors (two women) rated each single unicycle ride of the participants. First of all, raters watched all the videos of one session – without rating them – to get an overview of the range of unicycling proficiency. In a second run, they evaluated the unicycling performance on a five-point rating scale, ranging from 1 (very good) to 5 (poor). Unicycling-proficiency interrater agreement: ICC = 0.955. To enhance clarity in the presentation of the results, the scale of the unicycling performance was reverted so that higher scores (maximum of 5) reflect higher unicycling proficiency.

### Assessment of Postural control

Postural control was assessed at each time point of assessment (pre-test, post-test, follow-up) by means of an MFT Challenge Disc USB (TST Trendsport, Grosshöflein, Austria) which was connected to a laptop. The game-like software presented graded target rings and a small dot representing the position of the disc. Deviations/shifts of the center of gravity lead to movements of the dot, resulting in lower scores. Participants had to stand with both feet on the plate and tried to keep their equilibrium, i.e., to keep the dot as central as possible within the on-screen target for 20 s. To get a feeling for the plate and the software, participants were allowed to stay a few seconds on the balance board prior to the assessment of postural control^51^.

### MRI Data Acquisition

MRI data were acquired via a 3T Magnetom Skyra scanner (Siemens Healthineers, Erlangen, Germany) using a 32-channel head coil. Based on the Human Connectome Project^52^ we used a high resolution (0.7 mm isotropic) T1-weighted MPRage-Sequence to achieve highly accurate cortical surfaces and a good GM-WM contrast (TR = 2,400 ms, TI = 1,000 ms, TE = 2.32 ms, matrix = 320 × 320, FOV = 224 mm, 192 slices, thickness 0.7 mm, no gap, no PAT, FA = 8°). Diffusion-weighted images (DWI) were acquired with a multi-band accelerated EPI sequence protocol provided by the University of Minnesota Center for Magnetic Resonance Research (https://www.cmrr.umn.edu/multiband) (TR = 3,980 ms, TE = 110.40 ms, FoV = 256 mm, 69 slices, thickness 2 mm, no gap, no PAT, voxel size = 2 mm isotropic, 75 directions, b-value 1,000 s/mm^2^, multiband factor 3) with an anterior-posterior phase encoding direction. Additionally, one extra b0-image in the reverse phase-encoding direction was also acquired in order to correct for magnetic susceptibility-induced distortions.

MRI data of the control group were acquired on the same scanner and head coil but with partly different sequence parameters. Images were part of another project^28^, but the time-schedule was analogue to the current study. T1-weighted images were acquired with the same sequence (TR = 2,530 ms, TI = 900 ms, TE = 2.26 ms, matrix = 256 × 256, FOV = 256 mm, 176 slices, thickness 1 mm, voxel size 1 mm isotropic, gap = 0.5 mm, PAT = 2, FA = 9°). For diffusion weighted images a non-multiband EPI-sequence was used (TR = 8,900 ms, TE = 89 ms, FoV = 256 mm, 64 slices, thickness 2 mm, no gap, PAT = 2, voxel size = 2 mm isotropic, 64 directions, b-value 1,000 s/mm^2^).

### MRI Data Processing and Analysis

#### Longitudinal VBM and Cortical Thickness

First, intensity inhomogeneities of the T1-weighted images were corrected with the N4 bias field correction as provided by ANTs^53^. After visual inspection of the T1-weighted data, the following longitudinal VBM-analysis was processed using SPM12 (Wellcome Department of Cognitive Neurology, London, v6906) and the Computational Anatomy Toolbox (CAT12, r1113) under Matlab (The Mathworks, 2015). For each participant, all three bias-corrected images were affine co-registered to each other and the mean of the realigned images served as a reference image to the subsequent pre-processing steps. All realigned images were then segmented into GM, WM and cerebrospinal fluid tissue as implemented in SPM12. The deformation of the GM and WM images to MNI space was applied using the DARTEL-approach. For quality assurance we used the CAT12 toolbox to check the internal sample homogeneity. The mean correlation for all subjects was within two standard deviations. Finally, the spatially normalized and modulated GM images were smoothed with an isotropic kernel of 8 mm and served as inputs for the subsequent statistical analysis.

To examine changes in cortical thickness (CT) we used the surface-based morphometry approach newly implemented in the CAT12 toolbox. This fully automated method takes the segmented tissue classes (as already processed in the VBM-analysis) and uses a projection-based algorithm to compute CT^54^. Before smoothing the vertices with an isotropic kernel of 15 mm, we checked the internal sample quality of the surface data and had to exclude one subject.

An ANOVA for repeated measure was applied for the statistical analysis realized with the flexible factorial model in SPM including the factors time (pre, post and follow-up) and subject. Following post-hoc t-tests were computed: pre > post, pre > follow-up, post > follow-up and vice versa. All results were strictly corrected for multiple comparison using the FWE-correction at voxel-level with a p-value of 0.05.

#### Longitudinal DTI

First, raw DTI-images were visually checked for artifacts and then corrected for susceptibility-induced geometric distortions, subject motion and eddy current distortions using EDDY and TOPUP^55^. After computing a binary brain mask for each subject and time point, tensor-based matrices including fractional anisotropy (FA), mean diffusivity (MD), radial diffusivity (RD), axial diffusivity (AD) were computed with MRtrix^56^. Tract-based spatial statistics (TBSS, version 1,2) was used to conduct voxel-wise analysis^57,58^. FA images were aligned to the MNI standard space (1 mm isotropic) using a nonlinear registration. The mean FA image was then computed and afterwards thinned to create a mean FA skeleton. This skeleton is a representation of the centers of all common tracts of the group. A threshold of 0.2 was applied to restrict the analysis only to WM. The same procedure was carried out on all other non-FA tensor-based matrices including MD, RD and AD.

Changes in diffusion tensor image indexes were assessed with an ANOVA for repeated measure using a permutation-based non-parametric testing^59^ with 10,000 permutation followed by post-hoc t-tests analog to the VBM-statistics. Threshold-free cluster enhancement (TFCE) was applied with FWE to correct for multiple comparisons at a p-value of 0.05^60^. Statistical analyses for all metrices were restricted to the mean-skeleton mask.

#### Multivariate Pattern Recognition Analysis

We complemented our mass-univariate analysis by the use of multivariate pattern analysis provided by PRoNTo 2.0 (http://www.mlnl.cs.ucl.ac.uk/pronto) implemented in Matlab. Based on the pattern of whole-brain changes this analysis tries to predict a variable of interest (e.g., post-test – pre-test vs. follow-up – post-test). Due to the use of multivariate properties, this method can detect subtle changes in spatially distributed patterns greater sensitivity.

To investigate to which degree our complex balance training intervention changes the spatial distribution of GM and FA over time, we applied a linear support vector machine algorithm to the difference maps of the learning-phase (post-test – pre-test) and the post-learning-phase (follow-up – post-test). We used a leave-one-subject-per-group-out (LOSGO) cross-validation scheme to evaluate the model performance. Statistical significance of the classification accuracy was examined by nonparametric permutation testing (N_permutations_ = 10,000; *p* < 0.05). This analysis strategy was separately conducted for changes in GM volume, changes in FA and finally for both modalities combined.

Furthermore, to investigate the regional contribution to the discrimination function, an L1 multiple kernel learning algorithm was defined based on the Automatic Anatomic Labeling Atlas^61^ including 116 anatomical parceled regions of interest. The same cross-validation approach and statistical testing was used. This analysis was only conducted for GM maps.

### Control Analysis

All above-described analysis steps were also conducted for the control group with a few differences. For the control group only two time-points were available. As a consequence, the statistical test used was a paired t-test. Because of the missing third acquisition the MVPA could not be applied for the control group.

### Ethical statement

All methods and experiments were performed in accordance with relevant national and international guidelines and regulations and were approved by the local ethics committee. All subjects gave written informed consent to participate in this study.

## Supplementary information

Supplementary Information

## Data Availability

All data are available from the corresponding author upon reasonable request.

## References

[1] Azevedo FAC (2009). Equal numbers of neuronal and nonneuronal cells make the human brain an isometrically scaled-up primate brain. J. Comp. Neurol..

[2] Micheva KD, Busse B, Weiler NC, O’Rourke N, Smith SJ (2010). Single-Synapse Analysis of a Diverse Synapse Population: Proteomic Imaging Methods and Markers. Neuron.

[3] Nguyen, T. Total Number of Synapses in the Adult Human Neocortex. *Undergrad*. *J*. *Math*. *Model*. *One+Two* **3** (2013).

[4] Pascual-Leone A, Amedi A, Fregni F, Merabet LB (2005). The Plastic Human Brain Cortex. Annu. Rev. Neurosci..

[5] Maguire EA (2000). Navigation-related structural change in the hippocampi of taxi drivers. Proc. Natl. Acad. Sci..

[6] Mechelli A (2004). Structural plasticity in the bilingual brain. Nature.

[7] Stein M (2012). Structural plasticity in the language system related to increased second language proficiency. Cortex.

[8] Draganski B (2004). Changes in grey matter induced by training. Nature.

[9] Gaser C, Schlaug G (2003). Brain structures differ between musicians and non-musicians. J. Neurosci..

[10] Münte TF, Altenmüller E, Jäncke L (2002). The musician’s brain as a model of neuroplasticity. Nat. Rev. Neurosci..

[11] Langer N, Hänggi J, Müller NA, Simmen HP, Jäncke L (2012). Effects of limb immobilization on brain plasticity. Neurology.

[12] Bezzola L, Merillat S, Gaser C, Jancke L (2011). Training-Induced Neural Plasticity in Golf Novices. J. Neurosci..

[13] Hofstetter S, Tavor I, Tzur Moryosef S, Assaf Y (2013). Short-Term Learning Induces White Matter Plasticity in the Fornix. J. Neurosci..

[14] Johansen-Berg H, Baptista CS, Thomas AG (2012). Human Structural Plasticity at Record Speed. Neuron.

[15] Lakhani B (2016). Motor Skill Acquisition Promotes Human Brain Myelin Plasticity. Neural Plast..

[16] Taubert M (2010). Dynamic Properties of Human Brain Structure: Learning-Related Changes in Cortical Areas and Associated Fiber Connections. J. Neurosci..

[17] Taubert M, Lohmann G, Margulies DS, Villringer A, Ragert P (2011). Long-term effects of motor training on resting-state networks and underlying brain structure. Neuroimage.

[18] Thomas AG, Dennis A, Bandettini P (2012). a & Johansen-Berg, H. The effects of aerobic activity on brain structure. Front. Psychol..

[19] Johnson JA (1990). Unicycling. Nature.

[20] Ohsaki, H., Iwase, M., Sadahiro, T. & Hatakeyama, S. A consideration of human-unicycle model for unicycle operation analysis based on moment balancing point. In *2009 IEEE International Conference on Systems*, *Man and Cybernetics* 2468–2473, 10.1109/ICSMC.2009.5346351 (IEEE, 2009).

[21] Sehm B (2014). Structural brain plasticity in parkinson’s disease induced by balance training. Neurobiol. Aging.

[22] Ruffieux J (2018). Balance Training Reduces Brain Activity during Motor Simulation of a Challenging Balance Task in Older Adults: An fMRI Study. Front. Behav. Neurosci..

[23] Rogge AK (2017). Balance training improves memory and spatial cognition in healthy adults. Sci. Rep..

[24] Rogge A-K, Röder B, Zech A, Hötting K (2018). Exercise-induced neuroplasticity: Balance training increases cortical thickness in visual and vestibular cortical regions. Neuroimage.

[25] Taubert M, Mehnert J, Pleger B, Villringer A (2016). Rapid and specific gray matter changes in M1 induced by balance training. Neuroimage.

[26] Erickson KI (2011). Exercise training increases size of hippocampus and improves memory. Proc. Natl. Acad. Sci..

[27] Taubert M, Villringer A, Ragert P (2012). Learning-related gray and white matter changes in humans: An update. Neuroscientist.

[28] Fink A (2015). Training of verbal creativity modulates brain activity in regions associated with language- and memory-related demands. Hum. Brain Mapp..

[29] Halsband U, Lange RK (2006). Motor learning in man: A review of functional and clinical studies. J. Physiol..

[30] Hardwick RM, Rottschy C, Miall RC, Eickhoff SB (2013). A quantitative meta-analysis and review of motor learning in the human brain. Neuroimage.

[31] Ungerleider L (2002). Imaging Brain Plasticity during Motor Skill Learning. Neurobiol. Learn. Mem..

[32] Spampinato D, Celnik P (2017). Temporal dynamics of cerebellar and motor cortex physiological processes during motor skill learning. Sci. Rep..

[33] Boyke J, Driemeyer J, Gaser C, Buchel C, May A (2008). Training-Induced Brain Structure Changes in the Elderly. J. Neurosci..

[34] Bardiau M, Muylaert A, Duprez JN, Labrozzo S, Mainil JG (2010). Prevalence, molecular typing, and antibiotic sensitivity of enteropathogenic, enterohaemorrhagic, and verotoxigenic Escherichia coli isolated from veal calves. Tijdschr. Diergeneeskd..

[35] Gryga, M. *et al*. Bidirectional gray matter changes after complex motor skill learning. *Front*. *Syst*. *Neurosci*. **6** (2012). 10.3389/fnsys.2012.00037 PMC3353266 22623914

[36] Hüfner K (2011). Structural and functional plasticity of the hippocampal formation in professional dancers and slackliners. Hippocampus.

[37] Vaquero L (2016). Structural neuroplasticity in expert pianists depends on the age of musical training onset. Neuroimage.

[38] Aminoff EM, Kveraga K, Bar M (2013). The role of the parahippocampal cortex in cognition. Trends Cogn. Sci..

[39] Baier, B., Suchan, J., Karnath, H.-O. & Dieterich, M. Neural correlates of disturbed perception of verticality. *Neurology* (2012). 10.1212/WNL.0b013e318248e544 22357719

[40] Ellison A, Schindler I, Pattison LL, Milner AD (2004). An exploration of the role of the superior temporal gyrus in visual search and spatial perception using TMS. Brain.

[41] Karnath H-O, Ferber S, Himmelbach M (2001). Spatial awareness is a function of the temporal not the posterior parietal lobe. Nature.

[42] Karnath H-O (2001). New insights into the functions of the superior temporal cortex. Nat. Rev. Neurosci..

[43] Karnath H-O, Dieterich M (2006). Spatial neglect—a vestibular disorder?. Brain.

[44] Thiebaut de Schotten M (2005). Direct evidence for a parietal-frontal pathway subserving spatial awareness in humans. Science.

[45] Lunven M (2015). White matter lesional predictors of chronic visual neglect: A longitudinal study. Brain.

[46] Fryer SL (2008). Microstructural integrity of the corpus callosum linked with neuropsychological performance in adolescents. Brain Cogn..

[47] Navas-Sánchez FJ (2014). White matter microstructure correlates of mathematical giftedness and intelligence quotient. Hum. Brain Mapp..

[48] Thomas C, Baker CI (2012). Remodeling human cortex through training: Comment on May. Trends Cogn. Sci..

[49] Zatorre RJ, Fields RD, Johansen-Berg H (2012). Plasticity in gray and white: neuroimaging changes in brain structure during learning. Nat. Neurosci..

[50] Wenger E, Brozzoli C, Lindenberger U, Lövdén M (2017). Expansion and Renormalization of Human Brain Structure During Skill Acquisition. Trends Cogn. Sci..

[51] Hildebrandt C (2015). Functional assessments for decision-making regarding return to sports following ACL reconstruction. Part I: development of a new test battery. *Knee Surgery*. Sport. Traumatol. Arthrosc..

[52] Glasser MF (2013). The minimal preprocessing pipelines for the Human Connectome Project. Neuroimage.

[53] Avants BB (2011). NeuroImage A reproducible evaluation of ANTs similarity metric performance in brain image registration. Neuroimage.

[54] Dahnke R, Yotter RA, Gaser C (2013). Cortical thickness and central surface estimation. Neuroimage.

[55] Andersson JLR, Sotiropoulos SN (2016). An integrated approach to correction for off-resonance effects and subject movement in diffusion MR imaging. Neuroimage.

[56] Tournier J-D, Calamante F, Connelly A (2012). MRtrix: Diffusion tractography in crossing fiber regions. Int. J. Imaging Syst. Technol..

[57] Smith SM (2006). Tract-based spatial statistics: voxelwise analysis of multi-subject diffusion data. Neuroimage.

[58] Smith SM (2007). Acquisition and voxelwise analysis of multi-subject diffusion data with tract-based spatial statistics. Nat. Protoc..

[59] Nichols TE, Holmes AP (2002). Nonparametric permutation tests for functional neuroimaging: a primer with examples. Hum. Brain Mapp..

[60] Smith S, Nichols T (2009). Threshold-free cluster enhancement: Addressing problems of smoothing, threshold dependence and localisation in cluster inference. Neuroimage.

[61] Tzourio-Mazoyer N (2002). Automated Anatomical Labeling of Activations in SPM Using a Macroscopic Anatomical Parcellation of the MNI MRI Single-Subject Brain. Neuroimage.

