# Supplementary material for: Learning Unicycling Evokes Manifold Changes in Gray and White Matter Networks Related to Motor and Cognitive Functions
Source: Sci Rep. 2019 Mar 13;9:4324. doi: 10.1038/s41598-019-40533-6 (PMC6416294; doi:10.1038/s41598-019-40533-6)
Supplement: Supplementary file 1 — Supplementary Information [file 41598_2019_40533_MOESM1_ESM.pdf]

Supplementary Information

Learning Unicycling Evokes Manifold Changes in Gray and White Matter Networks Related to Motor- and Cognitive Functions

Weber, B., Koschutnig, K., Schwerdtfeger, A., Rominger, C., Papousek, I., Weiss, E. M., Tilp, M., & Fink, A.

Supplementary Table S1. Classification accuracy for changes in GM and FA

	GM	FA	GM & FA
Total accuracy (%)	80.43	50.00	78.26
p-value of balanced accuracy	0.002	0.462	0.001
Class accuracy (%) / p-value			
learning period	82.61 / 0.005	56.52 / 0.390	82.61 / 0.001
post-learning period	78.26 / 0.004	43.48 / 0.750	73.91 / 0.031

Table S1. The total classification accuracy to distinguish between learning period (post-test – pre-test) and post-learning period (follow-up – post-test) shows highest scores for GM. The balanced accuracy of the support vector machine analysis revealed a significant separation for the learning period using GM and a combination of GM and FA. Same is true for the post-learning period with higher p-values for GM. All results are based non-parametric permutation test ($N_{permutations}$ = 10.000).

Supplementary Figure S1. Multivariate pattern recognition analysis

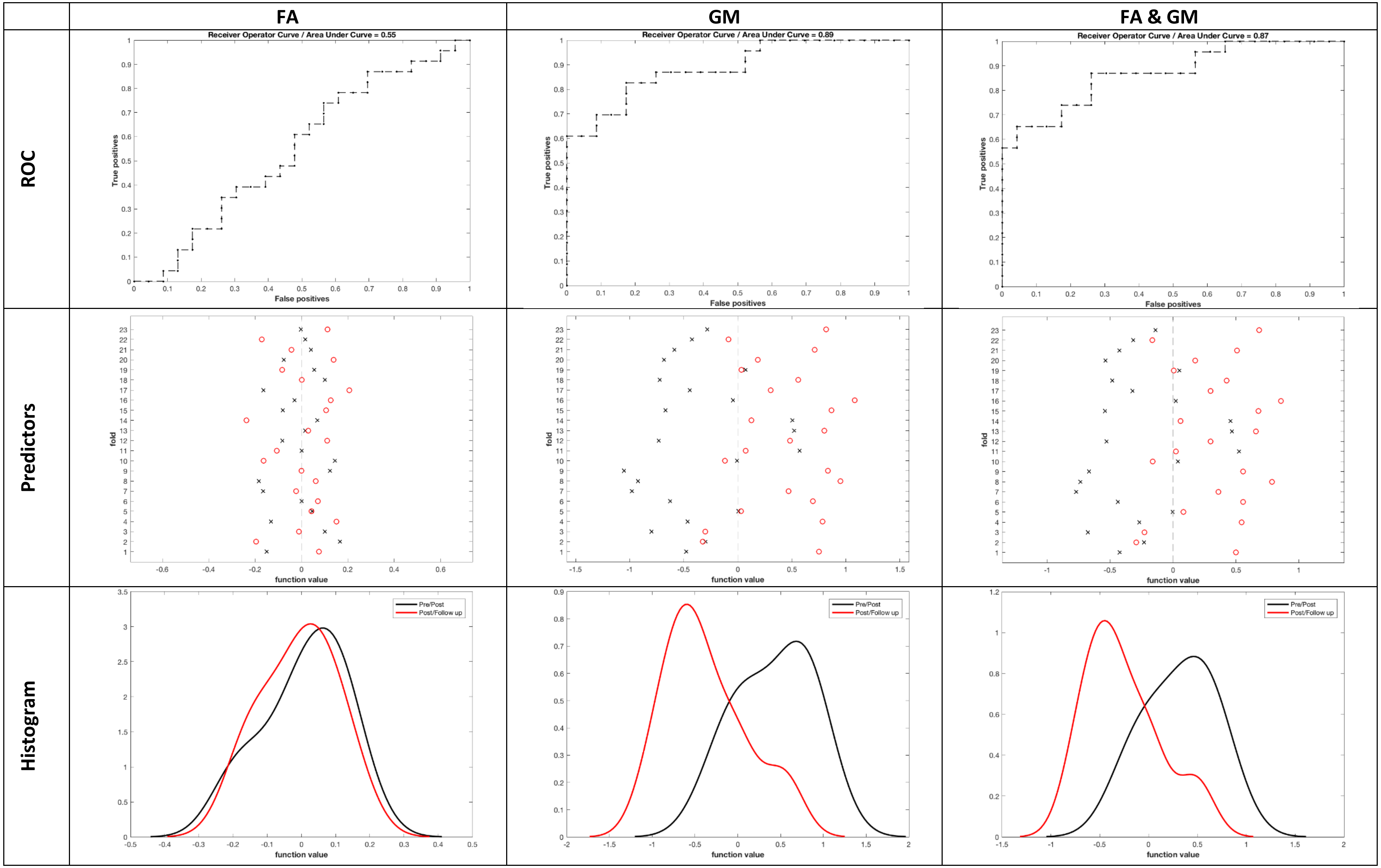

Figure S1 Support vector machine classification of the learning period and the post-learning period based on white-matter derived fractional anisotropy and GM volume. We performed a pattern recognition analysis for each modality separately and for both modalities together. As depicted in Supplementary Table S1 the highest classification accuracy has been found for GM volume changes The receiver operating characteristics (ROC) showing the tradeoff between specificity and sensitivity, including the area under the curve (0.55 for FA, 0.89 for GM and 0.87 for FA and GM). The highest classification accuracy for GM is supported by the plot of functional values, showing that for FA almost the half of the post-training values (red circles) are negative. For GM and for both modalities the majority of the post-training values have positive values. However, the difference between GM volume and both modalities is very small. This is supported by the corresponding histogram

Supplementary Figure S2. Changes in Axial and Mean Diffusivity

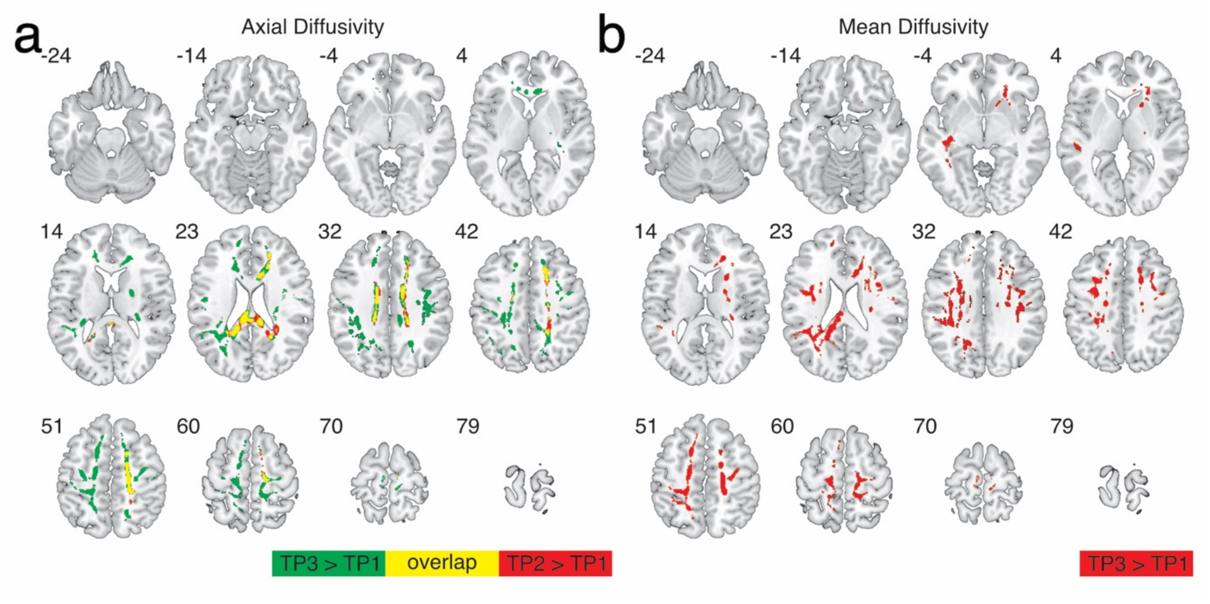

Figure S2. Axial (a) and mean diffusivity (b). Increases of axial diffusivity from TP1 to TP2 to TP3 and increases of mean diffusivity from TP1 to TP3. Results are FWE (p=0.05) corrected at voxel-level.

Supplementary Table S2. Changes in Axial and Mean Diffusivity

Contrast	Region	H	Cluster	1-p FWE	MNI-coordinates X	Y	Z
AD: post > pre							
	Corticospinal tract	R	9810	0.978	90	92	92
	Anterior thalamic radiation	L	52	0.954	106	139	119
AD: follow-up > pre							
	Forceps minor		2769	0.982	90	91	91
	Cingulum (cingulate gyrus)	L	1710	0.983	71	79	116
	Forceps major	R	182	0.959	67	75	95
	Superior longitudinal fasciculus	L	159	0.956	133	81	106
	Corticospinal tract	L	78	0.956	109	111	112
	Superior longitudinal fasciculus	L	33	0.953	130	71	112

MD: follow-up > pre						
Superior longitudinal fasciculus	L	4880	0.973	114	92	125
Superior longitudinal fasciculus	R	2403	0.972	70	106	121
Anterior thalamic radiation	R	1242	0.962	67	140	87
Forceps minor		413	0.958	73	155	90
Inferior fronto-occipital fasciculus	L	255	0.957	128	98	70
Superior longitudinal fasciculus	R	215	0.961	71	137	110
Superior longitudinal fasciculus	L	154	0.954	150	98	76
Inferior fronto-occipital fasciculus	L	104	0.953	103	163	108
Superior longitudinal fasciculus	L	97	0.954	100	143	128
Forceps minor		59	0.952	104	176	93

Table S2. Significant changes in DTI for AD and MD. (1-p)-values corrected for multiple comparison (FWE) are reported.
AD, axial diffusivity; MD, mean diffusivity; H, hemisphere; L, left; R, right; FWE, family-wise-error; pre, pre - test; post, post - test; follow-up, follow-up - test.
